# WRKY41/WRKY46-miR396b-5p-TPR module mediates abscisic acid-induced cold tolerance of grafted cucumber seedlings

**DOI:** 10.3389/fpls.2022.1012439

**Published:** 2022-09-08

**Authors:** Jin Sun, Jiaqi Chen, Xinyu Si, Weikang Liu, Mingzhu Yuan, Shirong Guo, Yu Wang

**Affiliations:** College of Horticulture, Nanjing Agricultural University, Nanjing, China

**Keywords:** abscisic acid, cold stress, cucumber, grafting, miR396b-5p, WRKY

## Abstract

Grafting is one of the key agronomic measures to enhance the tolerance to environmental stresses in horticultural plants, but the specific molecular regulation mechanism in this tolerance largely remains unclear. Here, we found that cucumber grafted onto figleaf gourd rootstock increased cold tolerance through abscisic acid (ABA) activating WRKY41/WRKY46-miR396b-5p-TPR (tetratricopeptide repeat-like superfamily protein) module. Cucumber seedlings grafted onto figleaf gourd increased cold tolerance and induced the expression of miR396b-5p. Furthermore, overexpression of cucumber miR396b-5p in Arabidopsis improved cold tolerance. 5’ RNA ligase-mediated rapid amplification of cDNA ends (5’ RLM-RACE) and transient transformation experiments demonstrated that *TPR* was the target gene of miR396b-5p, while *TPR* overexpression plants were hypersensitive to cold stress. The yeast one-hybrid and dual-luciferase assays showed that both WRKY41 and WRKY46 bound to *MIR396b-5p* promoter to induce its expression. Furthermore, cold stress enhanced the content of ABA in the roots and leaves of figleaf gourd grafted cucumber seedlings. Exogenous application of ABA induced the expression of *WRKY41* and *WRKY46*, and cold tolerance of grafted cucumber seedlings. However, figleaf gourd rootstock-induced cold tolerance was compromised when plants were pretreated with ABA biosynthesis inhibitor. Thus, ABA mediated figleaf gourd grafting-induced cold tolerance of cucumber seedlings through activating the WRKY41/WRKY46-miR396b-5p-*TPR* module.

## Introduction

Cucumber (*Cucumis sativus* L.) is one of the main vegetable crops in China, with the cultivation area reaching 1.27 million hm^2^ in 2020 (Lu L. et al., 2022). However, due to its weak tolerance and sensitivity to unsuitable environments, it often suffers from abiotic or biotic stresses, such as low temperature, high temperature, salt stress, downy mildew (*Pseudoperonospora cubensis*), and powdery mildew (*Podosphaera xanthii*) during the cultivation, which have become the limiting factors for the production of cucumber under protected cultivation (Das et al., 2022). Low temperature is a common environmental barrier factor in protected cultivation in China, which threatens the protected cultivation of cucumber in winter and spring (Feng et al., 2021). Grafting commercial cultivars with good quality, yield, and other traits onto strong rootstocks for cultivation is an effective way to overcome the stress damage of cucumber under low temperature (Li et al., 2021). At present, more than 30% of protected cucumber production in China adopts to grafting, which overcomes the low temperature barrier and effectively ensures the production of protected cucumber in low temperature growth seasons (Zhang H. M. et al., 2019). Previous studies revealed that the tolerance level of cucumber grafted seedlings to low temperature is largely dependent on the rootstock (Fu et al., 2021; Lv et al., 2022). Therefore, how to use the rootstock to regulate the cold tolerance of the scion is an important technical bottleneck for cucumber to achieve the grafting goal, and the core scientific problem behind it is how to obtain the cold tolerance of the scion from the rootstock.

Grafting functions by up-regulating a series of related genes involved in signal transduction, transcriptional regulation, protein expression, and antioxidant response (Li et al., 2014a,b). Furthermore, the scion microRNA (miRNA) and its target gene transcription level is regulated by grafting to adapt to environmental stress (Buhtz et al., 2010). miRNA is an endogenous non-coding RNA consisting of about 21–24 nucleotides. Mature miRNA binds to target mRNA by forming miRNA-induced silencing complex (RISC) and cutting or inhibiting translation, thereby achieving negative regulation of target genes and participating in the regulation of various physiological processes, such as cell division and differentiation, signal transduction, growth, and development, organ morphogenesis, and response to various biotic and abiotic stresses (Wang et al., 2017; Song et al., 2019; Li et al., 2022). Grafting alters the expression of miRNA and its target genes, indicating that miRNA plays an important role in regulating the physiological process of grafted seedlings at a post-transcriptional level (Khaldun et al., 2016; Li et al., 2016; Kehr and Kragler, 2018). In recent years, a large number of grafting-related miRNAs have been obtained by sRNA-Seq technology (Pagliarani et al., 2017; Yin et al., 2021; Hu et al., 2022). In our previous study on cucumber/pumpkin grafting compatibility, we isolated and identified 60 differentially expressed miRNAs in scions of grafted seedlings through high-throughput sequencing, but no specific miRNA was found (Ren et al., 2018), suggesting that the rootstock regulated a series of physiological characteristics mainly by inducing differential expression of miRNA inherent in the scion. Among the differentially expressed miRNAs, miR396b-5p was highly expressed in cucumber seedlings grafted onto figleaf gourd (*Cucurbita ficifolia* Bouché) (Ren et al., 2018). Figleaf gourd, a cold-tolerant species, has been widely used as the rootstock to improve cold tolerance of grafted cucumber (Zhou et al., 2009; Li et al., 2014b; Liu W. et al., 2021). However, it is not clear whether miR396b-5p mediates figleaf gourd-induced cold tolerance.

Grafting can change the endogenous hormone content of the scion through xylem transport of hormones, such as abscisic acid (ABA), salicylic acid, gibberellin, and cytokinin, thereby changing the physiological characteristics of the scion to adapt to the adverse environment (Zhang Z. et al., 2019; Fu et al., 2021; Guo et al., 2021), indicating that root hormones might play a vital role in root-shoot signal communication. ABA has been shown to be a signaling molecule that mediates communication between roots and shoots through activating H_2_O_2_ signaling, other hormones, or transcriptional regulatory pathways (Guo et al., 2021; Li et al., 2021; Lv et al., 2022). WRKY proteins, a family of plant-specific transcription factors, are involved in ABA-induced cold tolerance (Rushton et al., 2012). Exogenous application of ABA enhances the expression of four *WRKYs* in banana fruit during cold storage (Luo et al., 2017). Furthermore, cold stress induces the expression of *WRKY41* and *WRKY46* in cucumber, pepper (*Capsicum annuum*), and Pak-choi (*Brassica campestris* ssp. *chinensis*) (Wang et al., 2012; Zhang Y. et al., 2016; Wei et al., 2017), indicating that these two *WRKYs* might mediate cold tolerance. Indeed, ectopic overexpression of cucumber *WRKY46* in Arabidopsis enhances the tolerance to cold stress (Zhang Y. et al., 2016), while its regulation pathway is not well understood. Here, we found that cucumber grafted onto figleaf gourd enhanced the tolerance to cold stress as associated with the increase of ABA in roots and leaves. ABA induced the expression of *WRKY41* and *WRKY46* to further activate the transcript of *MIR396b-5p*, which directly cleaved the target gene of tetratricopeptide repeat-like superfamily protein (*TPR*), resulting in enhanced cold tolerance of grafted seedlings. Our results illustrated the molecular mechanism of miRNA-mediated source signal molecules that regulate the cold tolerance of grafted seedlings, which could guide the selection and use of cold-resistant rootstocks in the grafting cultivation and provide new ideas for the cultivation of new varieties of cold-resistant rootstocks.

## Materials and methods

### Plant materials and growth conditions

Cucumber (Jinchun No. 4) was used as scion, and figleaf gourd was used as rootstock in this study. The germinated seeds of cucumber and figleaf gourd as rootstocks were sown into 15-hole seedling trays containing commercial organic substrate (Jiangsu Xingnong Substrate Technology Co., Ltd., Zhenjiang, China). The cucumber seeds for the scion were sown after 3 d of rootstocks. Split grafts were performed when the cotyledons of cucumber (as scions) were fully expanded. The grafted plants with cucumber or figleaf gourd as rootstocks were designed as *Cs/Cs* or *Cs/Cf*, respectively. The plants were grown in a growth chamber and the growth conditions were maintained as follows: 22/18°C day/night, relative humidity of 65–75%, photosynthetic photon flux density (PPFD) of 300 μmol m^–2^ s^–1^, and a 14/10 h photoperiod.

### Experimental design

Experiment 1: To investigate the role of figleaf gourd rootstock on scion cold stress tolerance, the grafted plants with three true leaves were transferred into a growth chamber maintained at 4°C for cold treatment or maintained at 22°C for control treatment. The leaf samples were collected at 0 h, 6 h, 12 h, 24 h, 3 d, 5 d, and 7 d and, frozen in liquid nitrogen and stored at −80°C for further analysis.

Experiment 2: To test the effects of ABA on the cold tolerance of grafted plants, the *Cs/Cs* and *Cs/Cf* seedlings with three true leaves were divided into three groups: (1) In the control group, the *Cs/Cs* and *Cs/Cf* seedlings were watered with distilled water; (2) Exogenous ABA treatment, the *Cs/Cs* and *Cs/Cf* seedlings were watered with 100 μmol ABA; (3) Exogenous sodium tungstate (ST) treatment, ST, an inhibitor of molybdo-enzymes in plants, such as ABA aldehyde oxidase, is widely used as an ABA biosynthesis inhibitor in plants (Martin-Rodriguez et al., 2011; Carvajal et al., 2017; Jahan et al., 2021). To inhibit the biosynthesis of ABA, the Cs/Cs and Cs/Cf seedlings were watered with 1 mmol ST. For preparing the solution, ABA (Sigma-Aldrich, St. Louis, MO, United States) was dissolved in ethanol, and ST was dissolved in distilled water, then these solutions were diluted with distilled water at a ratio of 1: 10,000 (v:v). To exclude the influence of ethanol, an equal volume of ethanol was added into distilled water in the control treatment or ST solution in the ST treatment. Each group contained 24 plants and each plant was watered with 50 mL of solution. After watering for 24 h, half of the treated plants were exposed to cold stress at 4°C for 7 d, and the other plants were grown under normal conditions. The phenotype, maximum quantum yield of photosystem II (Fv/Fm), and the relative electrolyte leakage (REL) value were measured after cold stress for 7 d.

### Prediction of the target genes of miR396b-5p

The target genes of miR396b-5p were predicted using a plant miRNA target prediction server^1^ based on the expectation value ≤ 3 and the smaller unpair target site (UPE) value.

### 5’ RNA ligase-mediated rapid amplification of cDNA ends (5’ RLM-RACE)

5’ RLM-RACE was performed to verify the cleavage relationship of miR396b-5p to its predicted target genes using the FirstChoice™ RLM-RACE Kit (AM1700, Invitrogen, Carlsbad, CA, United States). The PCR products were cloned into a pMD-19T vector, and the clones were sequenced by General Biological Systems Co., Ltd., (Chuzhou, China).

### Construction of *MIR396b-5p* and *TPR* overexpression plants

The precursor of miR396b-5p was synthesized by General Biological Systems Co., Ltd., (Chuzhou, China), and ligated into the pCAMBIA1301 vector. The full-length CDS sequence of *TPR* was amplified using cucumber cDNA as the template with the specific primers (Supplementary Table 1), and inserted into the pFGC5941 vector. The constructed pCAMBIA1301-*MIR396b-5p* and pFGC5941-*TPR* plasmids were transformed into *Agrobacterium tumefaciens* strain EHA105, and Arabidopsis Col-0 wild-type (WT) plants were transformed using floral dip method (Clough and Bent, 1998). Transformed plants were selected and validated using qPCR, and the homozygous lines from T_3_ generations were used for cold stress with the same method of grafted cucumber seedlings.

### Yeast one-hybrid assay

The yeast one-hybrid assays were performed according to the methods as previously described (Wang et al., 2019). *MIR396b-5p* promoter sequence was amplified using the specific primers (Supplementary Table 1), inserted into the pAbAi vector, and linearized by *Bst*BI (Thermo Fisher Scientific, Rockford, IL, United States). The linearized vector was transferred into Y1HGold yeast strain. The full-length CDS sequences of cucumber *WRKY41* and *WRKY46* were amplified using the specific primers (Supplementary Table 1), and inserted into the pGADT7 vector. The pGADT7-*WRKY41*, pGADT7-*WRKY46*, or pGADT7 empty vector was transferred into the positive strain containing the bait vector, respectively, and cultured on the selection solid medium containing 110 ng mL^–1^ aureobasidin A (AbA) at 30°C for 3–5 d to detect DNA-protein interaction.

### Dual-luciferase assay

The dual-luciferase assays were performed according to the previous method (Yang et al., 2021). The CDS of *TPR* was amplified with the specific primers (Supplementary Table 1), and inserted into the pAC006 vector. The CDS of *WRKY41* and *WRKY46*, and the promoter sequence of *MIR396b-5p* were amplified and inserted into the pFGC5941-GFP and pGreenII 0800-LUC vectors, respectively, and transformed into *A. tumefaciens* strain GV3101 (pSoup-p19). The *A. tumefaciens* containing the indicated recombinant plasmid was injected into *Nicotiana benthamiana* leaves, and a Tanon 5200 Multi Image system (Tanon, Shanghai, China) was used to detect the luciferase luminescence after injected for 2 d. The firefly luciferase (LUC) and renilla luciferase (REN) activities were detected using the Duo-Lite™ Luciferase Assay System (Vazyme, Nanjing, China).

### RNA isolation and qPCR analysis

The miRNAs were isolated from each sample-treated with cold stress using a miRcute miRNA extraction Kit (Tiangen, Beijing, China) as previously described (Li et al., 2022). The isolated miRNA was reversely transcribed into cDNA using the Mir-X miRNA First-strand Synthesis Kit (Takara, Dalian, China). qPCR was performed using the TB Green Advantage qPCR Premix (Takara) with the specific primers (Supplementary Table 2). The *U6* gene was selected as an internal reference for standardized data.

Total RNA was extracted using a RNA simple Total RNA Kit (Tiangen), and reversely transcribed into cDNA using a HiScript II Q RT SuperMix Kit for qPCR (+ gDNA wiper) Kit (Vazyme). qPCR was performed using the TB Green^®^ Fast qPCR Mix (Takara) on a StepOnePlus™ Real-Time PCR System (Applied Biosystems, Foster, CA, United States) with the specific primers (Supplementary Table 2). The *actin* gene was selected as an internal reference for standardized data, and the relative gene expression level was calculated as the method previously described (Livak and Schmittgen, 2001).

### Measurement of biomass, proline, malondialdehyde content, REL, and Fv/Fm

The fresh and dry weight, proline and malondialdehyde (MDA) contents, REL as well as Fv/Fm were measured after cold stress for 7 d. The fresh and dry weight was measured with an electronic balance as previously described (Wang Y. et al., 2021). The content of proline was determined by the acidic ninhydrin colorimetric method (Bates et al., 1973). MDA content was measured by the thiobarbituric acid method (Hodges et al., 1999). The values of REL and Fv/Fm were determined according to previous methods (Zhang Y. et al., 2021).

### Determination of ABA content

The leaves or roots (0.2 g) of grafted seedlings were taken at 0 h, 6 h, 12 h, and 24 h of cold stress and determined with an ELISA Kit (Shanghai Renjie Biotechnology Co., Ltd.) as described by Li et al. (2022).

### Histochemical staining analysis of GUS

The CDS of *TPR*, or the promoter sequences of *WRKY41* and *WRKY46* were amplified using the specific primers (Supplementary Table 1), and inserted into the pBI121 vector. The recombinant plasmid was transformed into the *A. tumefaciens* strain GV3101. The tobacco was transiently transformed according to the previous method (Wang Y. et al., 2020). After transformation for 2 d, the leaves were stained by GUS staining Kit (Solarbio, Beijing, China) and photographed. For analyzing the role of ABA on the expression of *WRKY41* and *WRKY46*, tobacco leaves were subjected to foliar spray of 100 μmol ABA after injection for 1 d.

### Statistical analysis

All data were expressed as the mean ± SD (*n* = 3). The data were statistically analyzed using analysis of variance (ANOVA), and the significance of treatment differences was analyzed with Tukey’s test at *P* < 0.05 using SPSS 18.0 software (SPSS Inc., Chicago, IL, United States).

## Results

### Figleaf gourd rootstock improved cold tolerance of grafted cucumber

**FIGURE 1:**
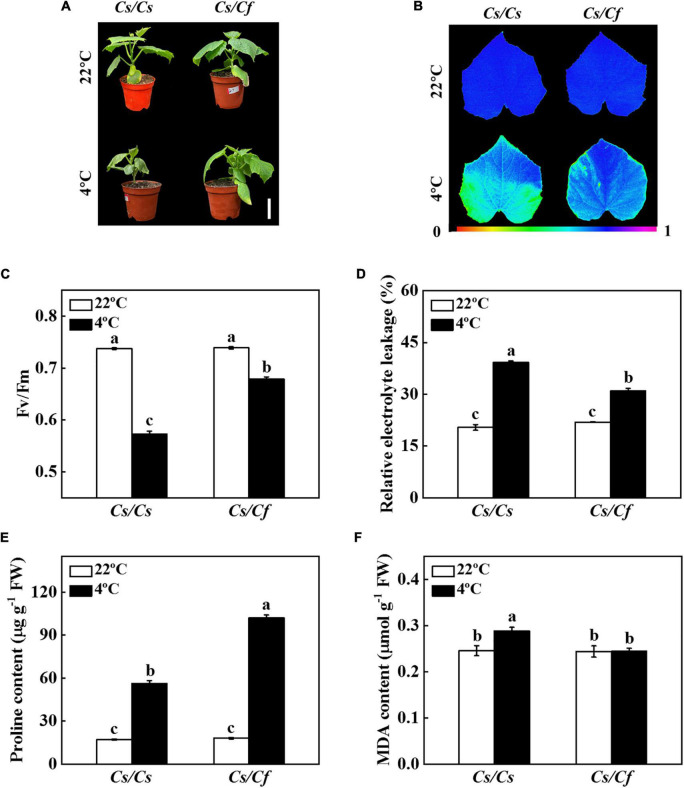
Figleaf gourd rootstock increased cold tolerance of grafted cucumber seedlings. **(A)** Plant phenotype under cold stress for 7 d. Bar: 5 cm. **(B,C)** The maximum quantum yield of photosystem II (Fv/Fm). **(D)** Relative electrolyte leakage (REL). **(E)** Proline content. **(F)** Malondialdehyde (MDA) content in leaves. Cucumber seedlings grafted onto cucumber (*Cs/Cs*) and figleaf gourd (*Cs/Cf*) were treated with cold stress at 4°C for 7 d, and the phenotype, Fv/Fm, REL, and MDA content were measured. The results represent the mean ± SD of 3 replicates. Means with the same letter did not significantly differ at *P* < 0.05 according to Tukey’s test. FW, fresh weight.

To analyze the cold tolerance of the grafted seedlings, the *Cs/Cs* and *Cs/Cf* plants were treated with cold stress for 7 d, and compared the phenotype, the values of Fv/Fm and REL, and the contents of proline and MDA. Cold stress induced the lower leaves wilting, a decrease of Fv/Fm value and significant increase of REL, proline, and MDA content in *Cs/Cs* and *Cs/Cf* plants (Figure 1). In contrast, figleaf gourd-grafted cucumber plants effectively ameliorated cold-induced leaf wilting, the decrease of Fv/Fm, and the increase of REL, and MDA content, as indicated that the value of Fv/Fm in *Cs/Cf* plants was 18.50% higher than that in *Cs/Cs* plants, and the level of REL and MDA content were significantly lower than that in *Cs/Cs* plants (Figure 1). These results indicated that cucumber grafted onto figleaf gourd significantly improved cold tolerance.

### Differentially expressed miR396b-5p mediated cold stress tolerance

**FIGURE 2:**
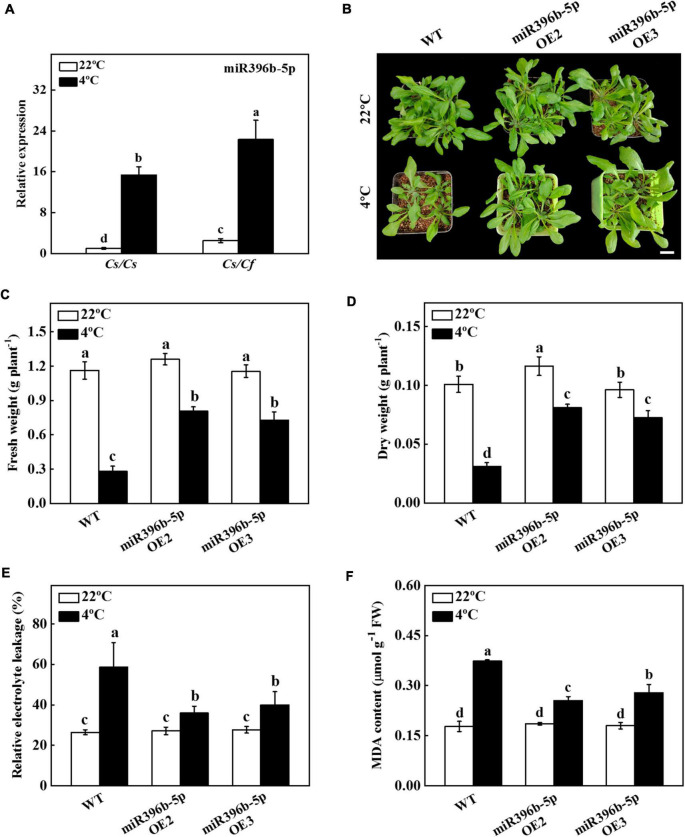
Functional analysis of miR396b-5p in response to cold stress. **(A)** The expression of miR396b-5p under cold stress. Cucumber seedlings grafted onto cucumber (*Cs/Cs*) and figleaf gourd (*Cs/Cf*) were treated with cold stress at 4°C for 24 h, and the expression of miR396b-5p was detected by qPCR. **(B)** Overexpression of cucumber miR396b-5p in Arabidopsis improved cold stress tolerance. Bar: 1 cm. **(C)** Fresh weight. **(D)** Dry weight. **(E)** Relative electrolyte leakage (REL). **(F)** Malondialdehyde (MDA) content in leaves. Arabidopsis seedlings were treated with cold stress at 4°C for 7 d, and the phenotype, fresh weight, dry weight, REL, and MDA content were measured. The results represent the mean ± SD (*n* = 3). Means with the same letter did not significantly differ at *P* < 0.05 according to Tukey’s test. FW, fresh weight.

In our previous study, we performed high-throughput sequencing on *Cs/Cs* and *Cs/Cf* plants and found that the expression of miR396b-5p in *Cs/Cf* plants was higher than that in *Cs/Cs* plants (Ren et al., 2018). It has been demonstrated that miR396, a conserved miRNA family, plays a vital role in plant growth and stress response (Yuan et al., 2020; Liu Y. et al., 2021). To investigate whether miR396b-5p mediated figleaf gourd-induced cold tolerance, we analyzed the expression level of miR396b-5p using qPCR under cold stress. As shown in Figure 2A, the expression level of miR396b-5p in *Cs/Cf* plants was 45.18% higher than that in *Cs/Cs* plants under normal growth conditions. Furthermore, cold stress significantly induced the expression of miR396b-5p, but its expression level in *Cs/Cs* plants was still significantly lower than that in *Cs/Cf* plants (Figure 2A). These results showed that miR396b-5p was induced by grafting and cold stress, suggesting that miR396b-5p might play an important role in figleaf gourd-induced cold tolerance.

To verify whether miR396b-5p played a critical role in the cold tolerance of grafted seedlings, we predicted the precursor sequence of miR396b-5p, and constructed miR396b-5p overexpression plants in *Arabidopsis thaliana* (miR396b-5p OE2 and miR396b-5p OE3). The expression level of miR396b-5p in miR396b-5p OE2 and miR396b-5p OE3 plants was 231.80-fold and 188.40-fold higher than that in WT plants (Supplementary Figure 1). After 7 d of cold stress treatment, WT plants grew slowly compared with miR396b-5p overexpression plants, and the fresh and dry weight of WT plants were significantly lower than those of miR396b-5p overexpression plants (Figures 2B–D). Further analysis of the physiological indicators of WT and miR396b-5p overexpression plants under cold stress showed that the value of REL of WT and miR396b-5p overexpression plants increased under cold stress, but the up-regulation level in WT plants was significantly higher than that in miR396b-5p overexpression plants (Figure 2E). The content of MDA in WT plants was 42.31% and 32.14% higher than that of the miR396b-5p OE2 and miR396b-5p OE3, respectively, after cold stress for 7 d (Figure 2F). These results indicated that overexpression of miR396b-5p could improve cold stress tolerance.

**FIGURE 3:**
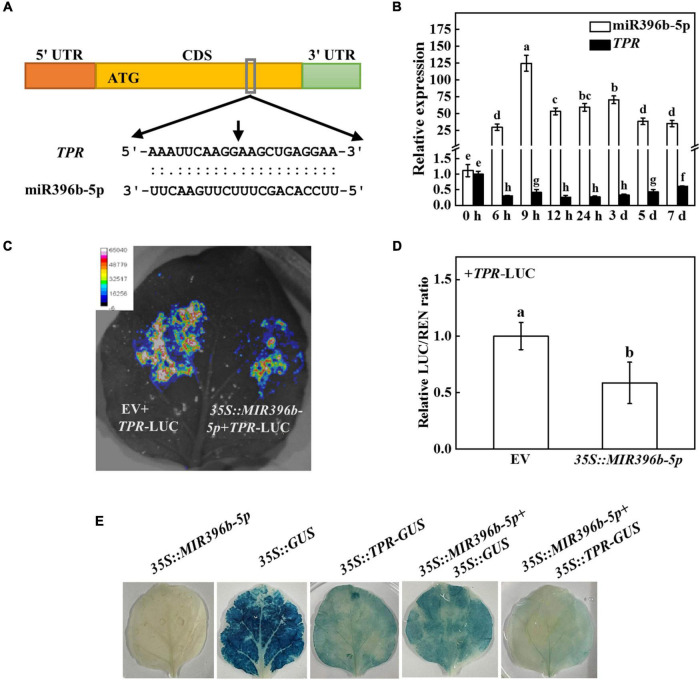
Cucumber *TPR* is the target gene of miR396b-5p. **(A)** 5’ RLM-RACE verified that there was a cleavage site in *TPR*, and the cleavage site is indicated by a black arrow. **(B)** Effects of cold stress on the expression of miR396b-5p and *TPR* in cucumber. Cucumber plants were exposed to cold stress and the leaves were harvested at the indicated time point for analysis the expression of miR396b-5p and *TPR* by qPCR. **(C,D)** Dual-luciferase assay verification of miR396b-5p cleavage of *TPR*. *Agrobacterium tumefaciens* harboring the indicated plasmids were infiltrated into tobacco leaves, and the leaves were analyzed after infiltration for 2 d. The empty vector was used as a control. The results represent the mean ± SD (*n* = 3). Means with the same letter did not significantly differ at *P* < 0.05 according to Tukey’s test. **(E)** GUS staining experiment verification of miR396b-5p cleavage of *TPR*. *A. tumefaciens* harboring the indicated plasmids were injected into tobacco leaves, and the leaves were stained after injection for 2 d. EV, empty vector.

In order to further investigate the mechanism of miR396b-5p under cold stress, we predicted the target genes by psRNATarget and found five target genes [CsaV3_6G012770, CsaV3_3G004170, CsaV3_2G030130, CsaV3_4G032160 (*TPR*), and CsaV3_6G045320], which were closely related to cold stress. To investigate whether the predicted target genes had a target-cleavage relationship with miR396b-5p, we used 5′ RLM-RACE to locate the miR396b-5p-directed cleavage site among the five predicted target genes. The results showed that a miR396b-5p-directed cleavage site was located in the 10th and 11th base pairs of the miR396b-5p target site in the CDS of *TPR* (Figure 3A). Interestingly, cold stress significantly induced the expression of miR396b-5p, but dramatically inhibited the expression of *TPR* (Figure 3B). The expression pattern of miR396b-5p and *TPR* was the opposite, indicating that *TPR* might be the target gene of miR396b-5p.

To further test whether *TPR* was the true target of miR396b-5p, we detected the interaction of miR396b-5p and *TPR* using luciferase and GUS staining experiment in tobacco leaves. As shown in Figure 3C, the fluorescence signal was weaker when co-injected with *TPR*-LUC and *35S:MIR396b-5p* than that co-injected with *TPR*-LUC and empty vector. Furthermore, the relative LUC/REN ratio in the leaves of co-inoculation of *TPR*-LUC and *35S:MIR396b-5p* was significantly lower than that in the control leaves (Figure 3D). GUS staining results showed that no blue staining was observed in the leaves inoculated with *35S:MIR396b-5p* overexpression vector (Figure 3E). When the leaves were injected with *35S:GUS* or *35S:TPR-GUS* or *35S:MIR396b-5p* and *35S:GUS*, GUS staining was observed with blue color in the injected leaves (Figure 3E). However, the blue color was lighter and the area was smaller when the leaves were co-injected with *35S:MIR396b-5p* and *35S:TPR-GUS* (Figure 3E). These results indicated that miR396b-5p negatively regulated the expression of *TPR* through direct cleavage.

### Overexpression of cucumber *TPR* in *Arabidopsis thaliana* reduced cold tolerance

To investigate the role of *TPR* in cold stress, we first analyzed its expression patterns in different tissues. Tissue expression analysis showed that *TPR* was expressed in different tissues, with less expression in roots, stems, and fruits, and higher expression in old leaves and mature leaves (Supplementary Figure 2). The expression level of *TPR* in old leaves was approximately 5 times that in mature leaves, and approximately 15 times that in tendrils and flowers (Supplementary Figure 2), suggesting that *TPR* was mainly expressed in cucumber leaves.

**FIGURE 4:**
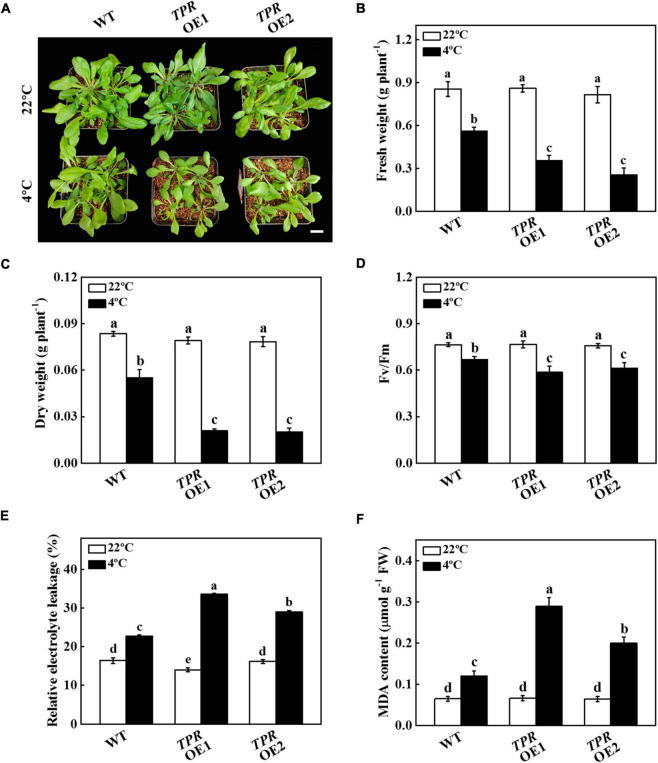
Functional analysis of *TPR* in response to cold stress. **(A)** Overexpression of cucumber *TPR* in Arabidopsis increased sensitivity to cold stress. Bar: 1 cm. **(B)** Fresh weight. **(C)** Dry weight. **(D)** The maximum quantum yield of photosystem II (Fv/Fm). **(E)** Relative electrolyte leakage (REL). **(F)** Malondialdehyde (MDA) content in leaves. Arabidopsis seedlings were treated with cold stress at 4°C for 7 d, and the phenotype, fresh weight, dry weight, Fv/Fm, REL, and MDA content were measured. The results represent the mean ± SD (*n* = 3). Means with the same letter did not significantly differ at *P* < 0.05 according to Tukey’s test. FW, fresh weight.

To further test the function of *TPR* under cold stress, we overexpressed cucumber *TPR* in Arabidopsis and obtained 2 independent overexpression plants (*TPR* OE1 and *TPR* OE2). The expression level of *TPR* gene in the two transgenic lines was detected by qPCR. As shown in Supplementary Figure 3, the expression level of cucumber *TPR* in WT plants was not detected, but its expression in *TPR* OE2 plants was 12.56 times that of *TPR* OE1 plants. *TPR* overexpression plants grew slower than WT plants during 7 d of cold stress treatment, as indicated by the fresh and dry weight of the *TPR* overexpression plants being significantly lower than that of the WT plants (Figures 4A–C). For instance, the fresh weight of *TPR* OE1 and *TPR* OE2 plants was 36.61% and 54.46% lower than that observed in WT plants, respectively (Figure 4B). The dry weight of *TPR* overexpression plants was 36.66–37.93% of WT plants (Figure 4C). Although cold stress significantly induced the decline of Fv/Fm, the value of Fv/Fm in WT was 12.26% and 8.37% higher than that in *TPR* OE1 and *TPR* OE2 plants, respectively, after 7 d of cold stress (Figure 4D). Further analysis of the physiological indicators of WT and *TPR* overexpression plants under cold stress showed that the value of REL of WT and *TPR* overexpression plants dramatically increased, but the increased level in the *TPR* overexpression plants was higher than that of WT plants (Figure 4E). In addition, the increased level of MDA content in *TPR* overexpression plants was significantly higher than that of WT plants after 7 d of cold stress treatment (Figure 4F), indicating that the cell membrane integrality of *TPR* overexpression was more damaged than that of WT plants under cold stress. These results indicated that overexpression of cucumber *TPR* reduced the tolerance to cold stress.

### Cucumber WRKY41 and WRKY46 bound to the promoter of *MIR396b-5p* to induce its expression

**FIGURE 5:**
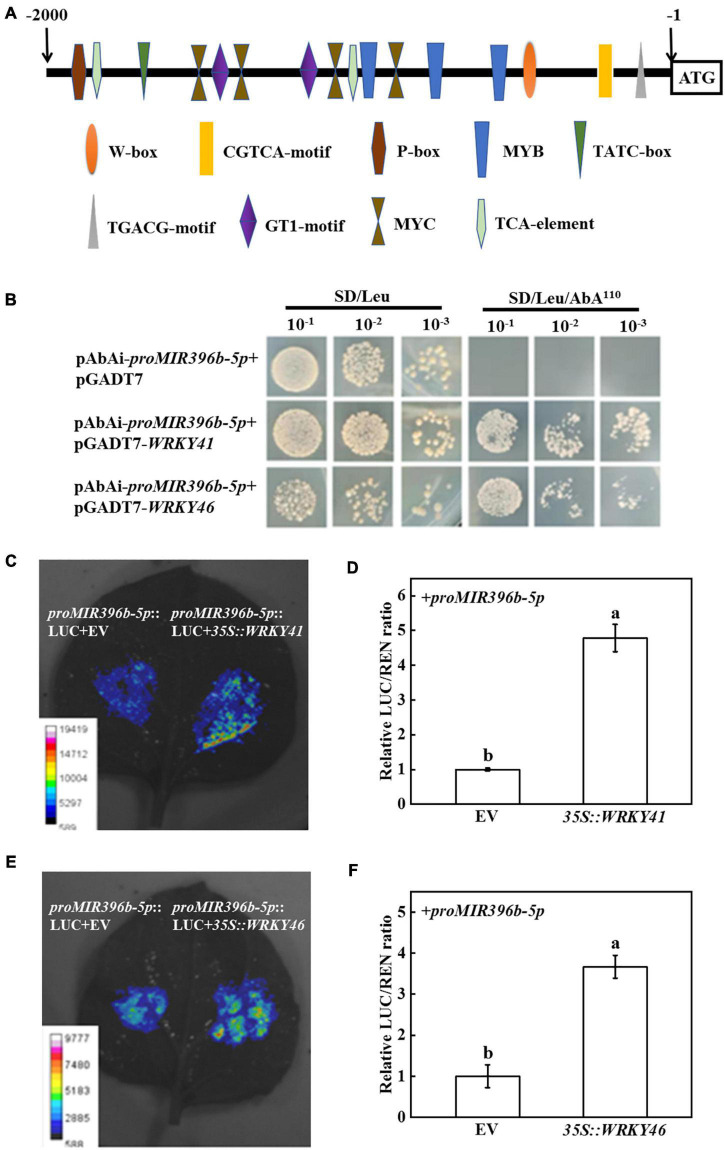
WRKY 41 and WRKY46 binding to the promoter of *MIR396b-5p*. **(A)** Analysis of the *cis-*acting elements in the promoter of *MIR396b-5p*. Numbering is from predicted transcriptional start sites. **(B)** Yeast one-hybrid assays indicating WRKY41 and WRKY46 binding to the promoter of *MIR396b-5p*. Yeast cells with DNA-protein interactions were grown on SD/Leu plates with 110 ng mL^–1^ aureobasidin A. **(C,D)** Dual-luciferase assay showing transient overexpression of *WRKY41* increase the expression of *MIR396b-5p* in *Nicotiana benthamiana* leaves. **(E,F)** Dual-luciferase assay showing transient overexpression of *WRKY46* increase the expression of *MIR396b-5p* in *N. benthamiana* leaves. *Agrobacterium tumefaciens* harboring the indicated plasmids were infiltrated into tobacco leaves, and the leaves were analyzed after infiltration for 2 d. The empty vector was used as a control. The results represent the mean ± SD (*n* = 3). Means with the same letter did not significantly differ at *P* < 0.05 according to Tukey’s test. EV, empty vector.

miR396b-5p participated in the response of grafted seedlings to cold stress by targeting and regulating the expression of *TPR* gene, but its upstream regulatory factor is unclear. To identify the transcription factors that regulate the expression of miR396b-5p, we first analyzed the promoter sequence of *MIR396b-5p* using the promoter analysis website PlantCare and PLACE. It was found that there were multiple *cis-*acting elements, such as W-box, MYB, and MYC, and hormone-responsive elements, including salicylic acid (TCA-element), methyl jasmonate (TGACG-motif, CGTCA-motif), gibberellin (P-box, TATC-box), in *MIR396b-5p* promoter sequence (Figure 5A).

Studies have shown that WRKY transcription factors can specifically interact with the *cis-*acting element of W-box, which activates or inhibits gene transcription to mediate the plant stress response (Yang et al., 2020; Wani et al., 2021; Rosado et al., 2022). Considering the promoter sequence of *MIR396b-5p* containing W-box, we screened WRKY transcription factors that bound to *MIR396b-5p* promoter using a yeast one-hybrid assay and found that yeast cells containing the bait vector of *MIR396b-5p* promoter sequence grew on SD/Leu medium containing 110 ng mL^–1^ AbA when transformed with pGADT7-*WRKY41* or pGADT7-*WRKY46* (Figure 5B). However, yeast cells containing the *MIR396b-5p* promoter sequence transformed with pGADT7 empty vector could not grow on the selection medium (Figure 5B). These results indicated that WRKY41 and WRKY46 bound to the promoter of *MIR396b-5p in vitro*. In order to confirm the interaction, we performed a dual-luciferase assay using tobacco transient transformation system. The fluorescence signals of tobacco leaves co-injected with *35S:WRKY41 or 35S:WRKY46* and *proMIR396b-5p*-LUC were stronger than those co-injected with empty vector and *proMIR396b-5p*-LUC (Figures 5C,E). Significantly, the relative LUC/REN ratio in the leaves co-injected with *35S:WRKY41 or 35S:WRKY46* and *proMIR396b-5p*-LUC was significantly higher than those co-injected with empty vector and *proMIR396b-5p*-LUC (Figures 5D,F). In addition, the expression levels of *WRKY41* and *WRKY46* in *Cs/Cf* plants were significantly increased under cold stress, among which, the expression level of *WRKY41* and *WRKY46* in *Cs/Cf* plants was 1.58-fold higher than that in *Cs/Cs* plants (Supplementary Figure 4). Therefore, transcription factors WRKY41 and WRKY46 in the figleaf gourd-grafted seedlings were up-regulated under cold stress, which promoted the expression of *MIR396b-5p* in cucumber leaves.

### ABA induced *WRKY 41* and *WRKY46* to regulate cold tolerance of grafted plants

**FIGURE 6:**
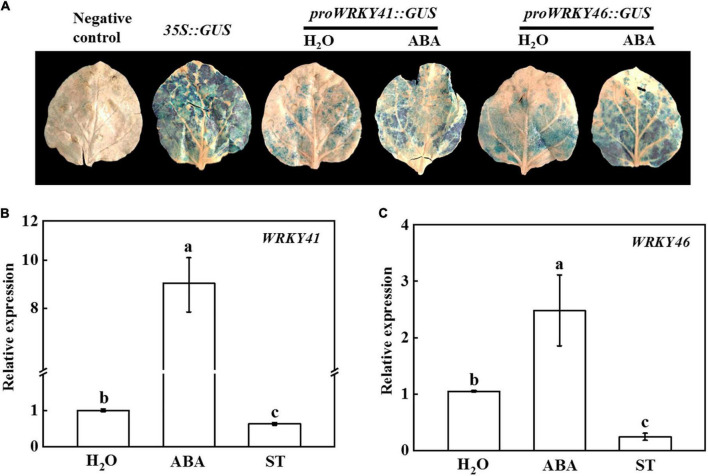
Effects of abscisic acid (ABA) on the expression of *WRKY41* and *WRKY46*. **(A)** Transient GUS expression in tobacco leaves showing ABA inducing the expression of *WRKY41* and *WRKY46*. *Agrobacterium tumefaciens* harboring the indicated plasmids were infiltrated into tobacco leaves, and the leaves were treated with 100 μmol ABA after infiltration for 1 d, and the GUS staining was performed after injection for 2 d. **(B)** Effects of ABA on the expression of *WRKY41* in cucumber leaves. **(C)** Effects of ABA on the expression of *WRKY46* in cucumber leaves. Cucumber plants were irrigated with 100 μmol ABA or 1 mmol sodium tungstate (ST), and the leaves were collected at 1 d for analysis the expression of *WRKY41* and *WRKY46* using qPCR. The results represent the mean ± SD (*n* = 3). Means with the same letter did not significantly differ at *P* < 0.05 according to Tukey’s test.

It has been verified that ABA plays important roles in grafted plants in response to cold stress (Guo et al., 2021; Lv et al., 2022). To test whether *WRKY41* and *WRKY46* could respond to ABA, we used tobacco transient transformation system to perform GUS staining experiment. As shown in Figure 6A, tobacco leaves injected with *proWRKY41:GUS* or *proWRKY46:GUS* detected slight blue staining, but the GUS staining was deeper and the area was bigger after foliar application of ABA. To further validate these results, cucumber seedlings were treated with exogenous water, ABA, or ST to analyze the expression of *WRKY41* and *WRKY46*. The results showed that compared with the control, the expression of *WRKY41* and *WRKY46* was significantly up-regulated by ABA treatment, while their expression was drastically blocked by ST (Figures 6B,C). Thus, the transcription levels of *WRKY41* and *WRKY46* were regulated by ABA.

**FIGURE 7:**
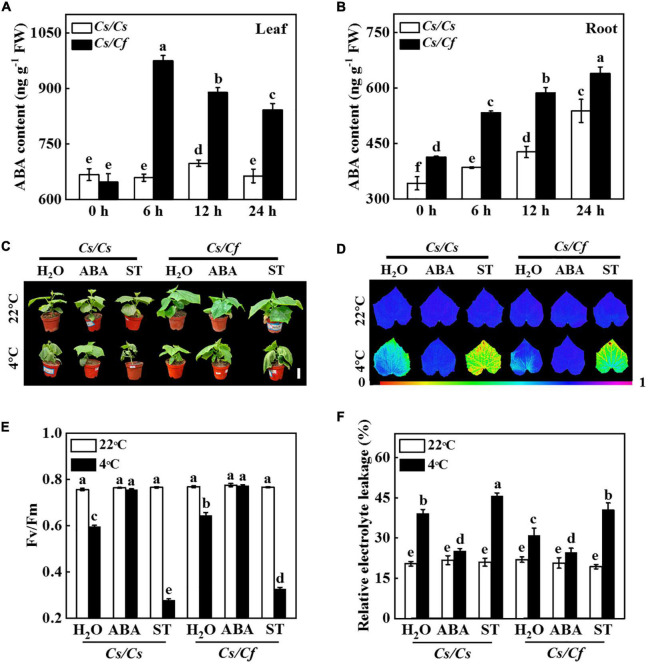
Abscisic acid (ABA) mediates figleaf gourd-induced cold stress tolerance. **(A)** ABA content in the leaves of grafted plants. **(B)** ABA content in the roots of grafted plants. Cucumber seedlings grafted onto cucumber (*Cs/Cs*) and figleaf gourd (*Cs/Cf*) were treated with cold stress at 4°C, and the leaves or roots were harvested at the indicated time points for analysis ABA content. **(C)** Phenotype of grafted plants treated with ABA or sodium tungstate (ST) under cold stress. **(D,E)** The maximum quantum yield of photosystem II (Fv/Fm) of grafted plants treated with ABA or ST under cold stress. **(F)** Relative electrolyte leakage (REL) of grafted plants treated with ABA or ST under cold stress. The *Cs/Cs* and *Cs/Cf* plants were irrigated with 100 μmol ABA or 1 mmol ST for 24 h, and then the plants were exposed to 4°C for 7 d to analyze the phenotype, Fv/Fm, and REL. The results represent the mean ± SD (*n* = 3). Means with the same letter did not significantly differ at *P* < 0.05 according to Tukey’s test. FW, fresh weight.

In order to investigate whether ABA mediated figleaf gourd-induced cold stress tolerance, we measured the dynamic changes of ABA content in leaves and roots of grafted seedlings under cold stress. The results showed that under cold stress, ABA content in leaves of *Cs/Cs* and *Cs/Cf* plants increased before 6 h and then decreased (Figure 7A). The content of ABA in the leaves of *Cs/Cf* plants reached the peak at 6 h and then decreased gradually, while *Cs/Cs* plants reached the peak at 12 h, but its ABA content was still significantly lower than that in *Cs/Cf* plants (Figure 7A). ABA content in *Cs/Cs* and *Cs/Cf* roots all significantly increased after cold stress and ABA content in *Cs/Cf* roots was significantly higher than that in *Cs/Cs* at any time point (Figure 7B). Therefore, figleaf gourd rootstock promoted the accumulation of ABA both in roots and leaves, which might play an important role in improving the cold tolerance of grafted seedlings.

To further test the effect of ABA on the response of grafted seedlings to cold stress, *Cs/Cs* and *Cs/Cf* plants were treated with cold stress after pre-irrigated exogenous ABA or its biosynthesis inhibitor ST. As shown in Figure 7C, the *Cs/Cs* plants-exposed to cold stress induced leaves wilting, but these effects were alleviated by pre-treatment with ABA. In contrast, when *Cs/Cs* plants were given pre-treatment with ST, the wilting was more serious than that pre-treatment with distilled water (Figure 7C). Strikingly, figleaf gourd rootstock-induced cucumber seedlings cold stress tolerance was compromised when plants were pretreated with ST (Figure 7C). Furthermore, the values of Fv/Fm and REL showed no significant difference in all of the plants under normal growth conditions (Figures 7D–F). However, cold stress significantly decreased the value of Fv/Fm in *Cs/Cs* plants, but the value of Fv/Fm in *Cs/Cs* plants pretreated with ABA was 26.85% higher than the plants pretreated with distilled water (Figures 7D,E). Furthermore, when *Cs/Cf* plants were pretreated with ST, their value of Fv/Fm was significantly lower than that in the distilled water-treated plants under cold stress (Figures 7D,E). Pretreatment with ABA significantly alleviated the decline of REL values both in *Cs/Cs* and *Cs/Cf* plants, but the values of REL in ST-pretreated plants were significantly higher than those of distilled water-treated plants under cold stress (Figure 7F). These results indicated that figleaf gourd rootstock-induced cold stress tolerance largely depended on ABA.

## Discussion

Grafting is one of the most commonly used techniques in vegetable crop production, which can improve stress tolerance, yield, and quality of vegetables (Shi et al., 2019; Wei et al., 2019; Darre et al., 2022). In recent years, many studies have shown that the cold tolerance of vegetable crops can be improved by grafting onto tolerant rootstock by maintaining higher photosynthesis, enhancing antioxidant activity to scavenge excess reactive oxygen species (ROS) (Xu et al., 2016; Lu J. et al., 2022; Luan et al., 2022), but information regarding the molecular mechanism of grafting in increasing cold tolerance remains largely unknown. In this study, we found that cucumber seedlings grafted onto figleaf gourd significantly increased the tolerance to cold stress through promoting the accumulation of ABA to induce the expression of *WRKY41* and *WRKY46*, which further activated the expression of miR396b-5b to target cleavage of *TPR*. Interestingly, cucumber grafted onto figleaf gourd not only promotes growth, but also increases the fruit yield (30%) in greenhouse during winter compared to non-grafted cucumber (Kumar et al., 2019), indicating that figleaf gourd is a promising rootstock for increasing cold tolerance of cucumber.

Numerous studies have reported that ABA participates in the regulation of grafted seedlings’ stress tolerance, such as cold, heat, salt, and drought stress (Shu et al., 2016; Niu et al., 2019; Zhang Z. et al., 2019; Habibi et al., 2022; Primo-Capella et al., 2022). A recent study using the cucumber/pumpkin grafting system showed that cold stress induces the increase of ABA content in leaves and roots of grafted seedlings, and ABA content positively correlates with the cold tolerance of varieties, and the increase of ABA content in leaves is mainly transportation from roots (Lv et al., 2022). Moreover, grafting of watermelon onto pumpkin increased the content of ABA in leaves and roots under cold stress, which was accompanied by a higher ABA exudation rate in the xylem (Guo et al., 2021). These results indicate that ABA acts as a long-distance signal molecule to participate in the cold stress response in grafted plants. Here, we found that cucumbers grafted onto figleaf gourds accumulated more ABA content both in leaves and roots in comparison to self-grafted plants under cold stress (Figures 7A,B). However, figleaf gourd-induced cold tolerance of cucumber seedlings was completely compromised by pre-treatment with an ABA biosynthesis inhibitor (Figures 7C–F). Therefore, ABA might function as a root-to-shoot signal to play an important role in improving the cold tolerance of grafting cucumber onto figleaf gourd. Indeed, rootstock-originated ABA is involved in grafting-induced cold stress tolerance through forming the feedback loop with melatonin and jasmonic acid (JA) (Guo et al., 2021). Moreover, ABA promotes the accumulation of H_2_O_2_ as the signal molecule to mediate rootstock-induced cold stress tolerance of grafting plants (Lv et al., 2022). It is worth noting that ABA activates numerous transcription factors, including WRKY, NAC, and bZIP, to mediate the plant stress response (Rushton et al., 2012; Ju et al., 2020; Liu C. et al., 2021; Wang T. J. et al., 2021).

WRKY, one of the largest families of transcription factors in higher plants, regulates plant growth, development, and stress responses (Ahammed et al., 2020; Li et al., 2020; Yang et al., 2020). For instance, overexpression of *WRKY12* from *Vitis amurens* in Arabidopsis or grapevine calli increases the tolerance to cold stress through enhancing the expression of genes related to antioxidants (Zhang L. et al., 2019). Studies have shown that overexpression of WRKY transcription factors induces the accumulation of ABA under abiotic stress (Ma et al., 2019; Dong et al., 2020). Furthermore, the ABA-responsive genes are significantly up-regulated in plants overexpressing *WRKYs* under drought and salt stress, indicating that *WRKY* regulates drought and salt tolerance of plants through ABA regulatory pathway (Dong et al., 2020; Huang et al., 2020). Conversely, ABA also induces the expression of *WRKYs* to mediate stress tolerance. Four WRKY transcription factors in banana fruit, including *WRKY31*, *WRKY33*, *WRKY60*, and *WRKY71*, were induced by exogenous ABA treatment under cold storage (Luo et al., 2017). Similarly, we found that the expression levels of *WRKY41* and *WRKY46* were significantly up-regulated under cold stress, which was accompanied by the increase of ABA content (Figures 7A,B and Supplementary Figure 4). Furthermore, *Agrobacterium*-mediated transient expression assays in tobacco leaves and qPCR analysis showed that exogenous ABA triggered the expression of *WRKY41* and *WRKY46*, but their expression was dramatically inhibited when cucumber leaves were treated with an ABA biosynthesis inhibitor (Figure 6). These results indicated that the transcription of *WRKY41* and *WRKY46* responded to ABA. Interestingly, overexpression of cucumber *WRKY46* in Arabidopsis increases cold stress tolerance as indicated by higher survival rates and proline content, less level of REL and MDA content, in comparison to WT plants (Zhang Y. et al., 2016). In addition, cucumber *WRKY46* overexpression plants enhance the sensitivity to ABA during seed germination and the expression of *ABI5* under cold stress (Zhang Y. et al., 2016). Therefore, ABA might induce the expression of *WRKY46*, which further strengthened ABA-dependent signal pathway to mediate the cold stress response.

Studies have shown that transcription factors can regulate the transcription of miRNA genes (Li and Yu, 2021; Li et al., 2022). In Arabidopsis, transcription factor ELONGATED HYPOCOTYL5 (HY5) binds to the promoter of *MIR775A* to inhibit its expression, resulting in negatively regulating the cell wall pectin level and cell wall elastic modulus to regulate organ size (Zhang H. et al., 2021). SQUAMOSA PROMOTER BINDING PROTEIN-LIKE (SPL) 9 can bind to the promoter of *MIR528* to activate the transcription of *MIR528* and plays an important role in rice antiviral response (Yao et al., 2019). In this study, it was found that cucumber WRKY41 and WRKY46 directly bound to *MIR396b-5p* promoter, and further transient transformation in tobacco proved that the transcription of *MIR396b-5p* was positively regulated by WRKY41 and WRKY46 (Figures 5B–F). Additionally, the expression levels of *WRKY41*, *WRKY46*, and *MIR396b-5p* were up-regulated when cucumber plants were exposed to cold stress (Figure 2A and Supplementary Figure 4). Thus, cold stress induced the expression of *WRKY41* and *WRKY46*, which bound to the promoter of *MIR396b-5p* to activate its transcription to form a mature miR396b-5p. miRNA-mediated regulation of target genes is a critical mechanism of plants response to cold stress. Overexpression of miR319 increases cold tolerance in rice (*Oryza sativa* L.) and sugarcane (*Saccharum officenarum* L.) through targeting TCP transcription factors (Thiebaut et al., 2012; Yang et al., 2013). miR393 mediates cold stress tolerance in switchgrass (*Panicum virgatum* L.) *via* down-regulating the expression of auxin receptor gene (Liu et al., 2017). Overexpression of rice miR1320 enhances cold tolerance by targeting an ERF transcription factor, ERF096, to activate the JA-mediated cold signal pathway (Sun et al., 2022). miR396, a conserved miRNA family in plants, has been confirmed to be involved in regulation of plant growth and development as well as abiotic stress responses (Liebsch and Palatnik, 2020; Liu Y. et al., 2021; Pegler et al., 2021). The miR396-GRFs module regulates the brassinosteroid-mediated prevention of photo-oxidative damage of etiolated seedlings exposed to light in Arabidopsis (Wang L. et al., 2020). Overexpression of rice miR396c in creeping bentgrass (*Agrostis stolonifera*) inhibits growth, but increases salt tolerance as observed by higher chlorophyll content, cell membrane integrity, and a decrease in the accumulation of Na^+^ (Yuan et al., 2019). Ectopic overexpression of tomato miR396a-5p in tobacco significantly increases cold stress tolerance *via* decrease the production of ROS (Chen et al., 2015). miR396b of trifoliate orange (*Poncirus trifoliate*) positively enhances cold tolerance by cleavage of *ACO*, inhibiting ethylene synthesis and promoting polyamine synthesis, resulting in increasing the activity of ROS scavenging (Zhang X. et al., 2016). Here, we found that cucumber miR396b-5p improved cold tolerance through targeting *TPR* (Figures 2, 3), indicating the conserved role of miR396 in the response to plant stress.

Cucumber TPR was a tetratricopeptide repeat-like (TPL/TPR) superfamily protein. TPL/TPR family proteins are widely present in plants and participate in the regulation of plant growth and development, stress response, signaling pathway regulation, and other physiological and biochemical activities (Sharma and Pandey, 2016). TPL/TPR can stably interact with specific transcriptional inhibitors to inhibit the expression of target genes (Gallavotti et al., 2010). Studies have shown that TPL/TPR protein plays critical roles in hormone signaling pathways (Lin et al., 2009; Fukazawa et al., 2021). ETO1, a repressor in the ethylene signal pathway, contains the TPR motifs in the C-terminus to interact with ACS5 to promote its degradation *via* 26S proteasome in Arabidopsis (Wang et al., 2004; Yoshida et al., 2005). SRFR1 encodes a TPR protein and acts as a suppressor in effector-triggered immunity (Kwon et al., 2009). In this study, we found that *TPR* was predominantly expressed in cucumber leaves, and its expression was blocked under cold stress (Figure 3B and Supplementary Figure 2). Moreover, *TPR* overexpression plants were more sensitive to cold stress compared with WT plants (Figure 4). These results indicated that cucumber TPR was a negative regulator during cold stress, but its specific function mechanism was still unknown.

**FIGURE 8:**
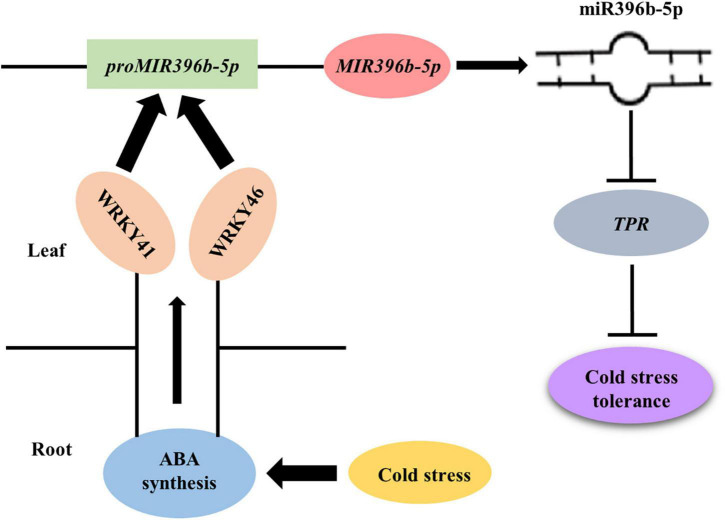
A proposed model for the molecular regulatory pathway of cucumber seedling grafted onto figleaf gourd rootstocks. When grafted cucumber seedling exposed to cold stress, ABA accumulated in roots and leaves, which induced the expression of *WRKY41* and *WRKY46*. Then, WRKY41 and WRKY46 bound to the promoter of *MIR396b-5p* to activate its transcription to form mature miR396b-5p. miR396b-5p negatively regulated the target gene *TPR via* direct cleavage, resulting in improving the cold tolerance of grafted seedlings.

In conclusion, we have provided a molecular regulatory pathway of figleaf gourd grafting-induced cold stress tolerance (Figure 8). When figleaf gourd grafted cucumber seedlings were exposed to cold stress, ABA accumulated both in roots and leaves. Then, ABA induced the expression of *WRKY41* and *WRKY46*, which further bound to the promoter of *MIR396b-5p* to promote its transcription. miR396b-5p negatively regulated the expression of *TPR*, thereby enhancing the cold tolerance of grafted seedlings. These findings reveal the downward signal pathway of ABA-mediated grafting cold tolerance. However, how ABA induces the expression of *WRKY41* and *WRKY46* under cold stress needs further investigation.

## Data availability statement

The original contributions presented in this study are included in the article/Supplementary material, further inquiries can be directed to the corresponding author/s.

## Author contributions

YW and JS designed the experiment. JS, JC, XS, WL, and MY performed experiments, among which JS, JC, and XS performed most of the experiments and analyzed the data. SG analyzed the data. YW and JS wrote the original draft. YW, JC, and XS revised the manuscript. All authors contributed to the article and approved the submitted version.

## Footnotes

1
http://plantgrn.noble.org/psRNATarget/
