# Supplementary material for: WRKY41/WRKY46-miR396b-5p-TPR module mediates abscisic acid-induced cold tolerance of grafted cucumber seedlings
Source: Front Plant Sci. 2022 Sep 8;13:1012439. doi: 10.3389/fpls.2022.1012439 (PMC9493262; doi:10.3389/fpls.2022.1012439)
Supplement: Supplementary file 1 [file Data_Sheet_1.PDF]

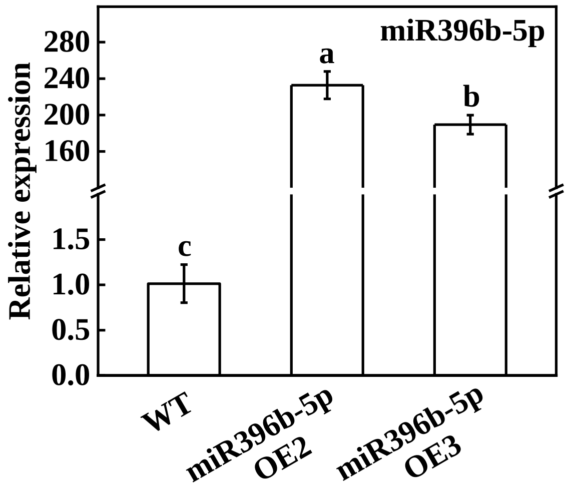

SUPPLEMENTARY FIGURE 1. Relative expression level of miR396b-5p in transgenic Arabidopsis plants. The results represent the mean ± SD of 3 replicates. Means with the same letter did not significantly differ at $P < 0.05$ according to Tukey's test.

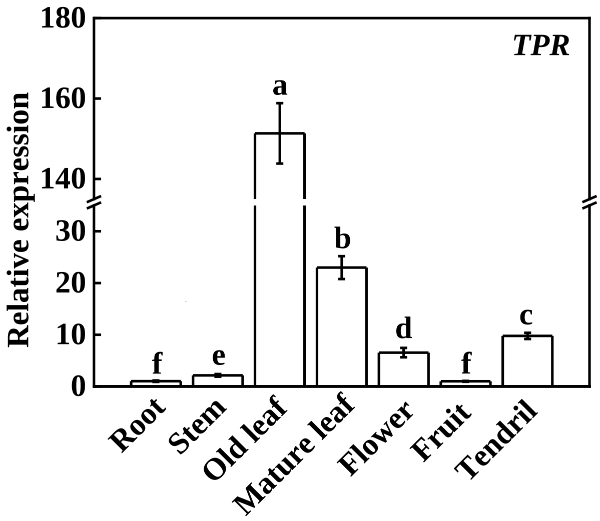

SUPPLEMENTARY FIGURE 2. Expression patterns of *TPR* in different tissues of cucumber. qPCR analysis the expression of *TPR* in root, stem, old leaf, mature leaf, flower, fruit, and tendril of cucumber. The expression level in root was set to 1.0. The results represent the mean ± SD of 3 replicates. Means with the same letter did not significantly differ at $P < 0.05$ according to Tukey's test.

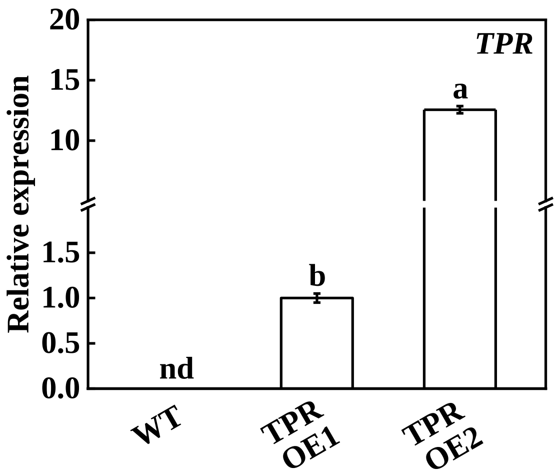

SUPPLEMENTARY FIGURE 3. Relative expression level of *TPR* in transgenic Arabidopsis plants. The results represent the mean ± SD of 3 replicates. Means with the same letter did not significantly differ at $P < 0.05$ according to Tukey's test. nd, none detected.

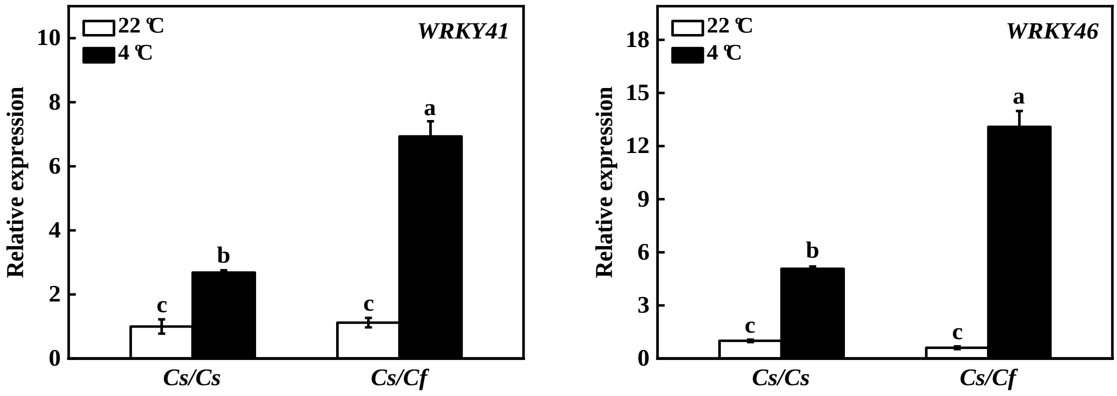

SUPPLEMENTARY FIGURE 4. Cold stress induced the expression of *WRKY41* and *WRKY46* in grafted cucumber plants. Cucumber seedlings grafted onto cucumber (*Cs/Cs*) and figleaf gourd (*Cs/Cf*) were treated with cold stress at 4°C, and the leaves were harvested at 24 h for analysis the expression of *WRKY41* and *WRKY46*. The results represent the mean ± SD of 3 replicates. Means with the same letter did not significantly differ at $P < 0.05$ according to Tukey’s test.
