# Supplementary material for: WRKY41/WRKY46-miR396b-5p-TPR module mediates abscisic acid-induced cold tolerance of grafted cucumber seedlings
Source: Front Plant Sci. 2022 Sep 8;13:1012439. doi: 10.3389/fpls.2022.1012439 (PMC9493262; doi:10.3389/fpls.2022.1012439)
Supplement: Supplementary file 2 [file Data_Sheet_2.PDF]

Supplementary Table 1. Primer sequences for vector construction.

Primer name	Forward primer	Reverse primer
pFGC5941-*TPR*	5'-tacaaatctatctctctcgagATGCCTCAAACTCTAATTCTGAAGC-3'	5'-ggatccccgggtaccgagctcGCAAATCTTAGCGCATAAACCC-3'
pAbAi-*proMIR396b-5p*	5'-cttgaattcgagctcggtaccTCTTTCAATTTCTTTATTATTTCTTCTGC-3'	5'-atacagagcacatgcctcgagTTCAAATTAACAAATTATATGATCATATAAAGA-3'
pGADT7-*WRKY41*	5'-gccatggaggccagtgaattcATGGAAAGCGGGTGGAGC-3'	5'-cagctcgagctcgatggatccTTAGTAGAAAAATCCTTGGGAATTAAATGG-3'
pGADT7-*WRKY46*	5'-gccatggaggccagtgaattcATGTCGGATGAAATGTTTAAAGATTT-3'	5'-cagctcgagctcgatggatccTCACGGCTGTCGGTTGAAAAACG-3'
pAC006-*TPR*	5'-ggaaagatcgccgtgggatccTAAGGAACTTTAGTGAAATGGATTTCG-3'	5'- actctagggactagtcccgggTTCAAAAGACATCAAACTATTATAAGTAACTG-3'
pGreenII0800-*proMIR396b-5p*	5'-ctatagggcgaattgggtaccTCTTTCAATTTCTTTATTATTTCTTCTGC-3'	5'-atcgataccgtcgacctcgagAAGCATGGCTATGACCTGAAAAA-3'
pFGC5941-*WRKY41*	5'-tacaaatctatctctctcgagATGGAAAGCGGGTGGAGC-3'	5'-ggatccccgggtaccgagctcGTAGAAAAATCCTTGGGAATTAAATGG-3'
pFGC5941-*WRKY46*	5'-tacaaatctatctctctcgagATGTCGGATGAAATGTTTAAAGATTT-3'	5'-ggatccccgggtaccgagctcCGGCTGTCGGTTGAAAAACG-3'
pBI121-*proWRKY41*	5'-gaccatgattacgccaagcttCTATAATGGTGGTGGTGTGCAAA-3'	5'-ggactgaccacccggggatccTTTTAATTCCTTTCAGTTCATAAAATTG-3'
pBI121-*proWRKY46*	5'-gaccatgattacgccaagcttGCTCTGTTTAGGTGTAATAAAAATTAA-3'	5'-ggactgaccacccggggatccCGTTTATCAATCTCGAAAAACGAC-3'
pBI121-*TPR*	5'-acgggggactctagaggatccATGCCTCAAACTCTAATTCTGAAGC-3'	5'-gcccttgctcaccatggtaccGCAAATCTTAGCGCATAAACCC-3'

Supplementary Table 2. Primers used for qPCR assays.

Gene name	Forward primer	Reverse primer
U6	5'-GGGGACATCCGATAAAATT-3'	5'-TGTGCGTGTCATCCTTGC-3'
miR396b-5p	5'-TTCCACAGCTTTCTTGAACTT-3'	
TPR	5'-TTGGTGGTTACCTGTATG-3'	5'-ATTCTTCTGCTCTGCCTC-3'
WRKY41	5'-CCCGATAAGCAAGAACAG-3'	5'-GAGCCAAAGCAGAAGGTA-3'
WRKY46	5'-AAGAACAGTGCTTATCCCAG-3'	5'-AGTCGCCGGAATTAGGTG-3'
CsActin	5'-CAGGAATCCACGAAACTACT-3'	5'-AGACCCTCCAATCCAAACAC-3'
AtActin	5'-GGGGACATCCGATAAAATT-3'	5'-TGTGCGTGTCATCCTTGC-3'
